# Projected effect of a high performing blood test on wait times for determination of Alzheimer's disease pathology in the United States

**DOI:** 10.1177/13872877261467345

**Published:** 2026-07-16

**Authors:** Soeren Mattke, Jiahe Chen, Mark Hanson, Kim G. Johnson, Cara Leahy, David A. Merrill, Victoria Shada, Jorge G. Ruiz

**Affiliations:** 1 The USC Brain Health Observatory, USC Dornsife, Los Angeles, CA, USA; 2 Department of Neurology, 3065 Duke University School of Medicine, Durham, NC, USA; 3 Memorial Healthcare Institute for Neuroscience, Owosso, MI, USA; 4 Pacific Brain Health Center, 731790 Pacific Neuroscience Institute, Saint John's Health Center, Santa Monica, CA, USA; 5 Department of Internal Medicine, Section on Gerontology and Geriatric Medicine, 12279 Wake Forest School of Medicine, Winston-Salem, NC, USA; 6 3933 Memorial Healthcare System, Hollywood, FL, USA

**Keywords:** Alzheimer's disease, amyloid-targeting treatments, blood biomarker, clinical utility, health system capacity, wait times

## Abstract

**Background:**

Blood tests for Alzheimer's disease (AD) are emerging as alternative to amyloid positron emission tomography (PET) scans and analysis of cerebrospinal fluid (CSF). However, their effect on clinical decision-making and subsequent wait times in the diagnostic process remain unclear.

**Objective:**

To estimate wait times in the diagnostic process to determine potential eligibility for amyloid-targeting treatments.

**Methods:**

We used a structured expert consultation process to elicit how a blood test to rule out or to confirm AD pathology would inform decisions in primary and secondary care and a Markov model to project how those decisions would affect wait times for specialist visits and amyloid PET scans.

**Results:**

Compared with referral decisions based on the results of a brief cognitive assessment, the addition of an AD blood test is projected to reduce wait times for subsequent specialist appointments by 35%. A blood test for confirmation of AD pathology would also eliminate wait times for amyloid PET scans.

**Conclusions:**

High-performing AD blood tests have the potential to streamline the diagnostic journey and reduce wait times

## Introduction

The emergence of treatments^1,2^ that target the pathobiology of Alzheimer's disease (AD) rather than its symptoms represents a paradigm shifting event in two ways. The approval, initially in the U.S. and later in other jurisdictions, of three amyloid-targeting treatments provided support for the brain amyloid hypothesis and gave new hope to patients and their families. However, it also changed the nature of memory care, which had historically focused on symptom management and social care rather than the highly medicalized nature of amyloid-targeting treatments. Put differently, the long-standing absence of a treatment meant the absence of a robust treatment ecosystem for diagnosis, infusion delivery and monitoring.

The concerns about health systems’ readiness to identify patients, who are eligible for amyloid-targeting treatments, and administer and monitor their treatment emerged soon after a 2016 publication^3^ showed that aducanumab, one of those treatments, was able to remove amyloid deposits and decelerate the progression of the disease. In 2017, Ritchie and colleagues released the Edinburgh Consensus,^4^ in which they laid out the requirements for delivering those amyloid-targeting treatments and cautioned that health systems even in high-income countries were not prepared for that new task. In the same year, a modeling study predicted substantial wait times, particularly for appointments with memory specialists in the United States (US)^5^ and later in other countries.^6^

The early experience in the US, where amyloid-targeting treatments have been available since 2021, provides anecdotal evidence for long wait times in the diagnostic process.^7^ However, the root cause does not seem to be overwhelming demand but a much slower than expected engagement of brain health specialists: by 2023, only 924 patients in traditional Medicare had started treatment and only 470 unique prescribers accounted for those patients.^8^

Several other assumptions in the initial predictions proved incorrect. The first is that mild cognitive impairment (MCI) would be reliably detected in primary care and lead to further evaluation in specialty care, whereas a 2023 study^9^ suggested that only 7% of expected MCI cases in the Medicare population aged 65 and older were actually diagnosed. The second is that referrals into specialty care and subsequent diagnostic steps were based on test results alone, which failed to account for the complexity of shared decision-making in this elderly and multimorbid population, and considerations like competing mortality risks, relative contraindications and patient preferences are important factors to consider.^10,11^ Third, prior studies did not account for the slow diffusion of new technologies and treatments in medicine.^12^

Lastly, the emergence of high-performing blood tests for AD pathology, which begin to rival the accuracy of amyloid positron emission tomography (PET) and cerebrospinal fluid (CSF) analysis,^13^ has the potential to streamline the process of determining potential eligibility for amyloid-targeting treatments, thus reducing wait times. Against this background, the current study aims to generate updated estimates for wait times because of constraints in diagnostic capacity, taking into account the effect of test results on referral decisions, speed of diffusion, and the impact of blood tests.

## Methods

### Overview

We used a Markov model to predict wait times in the diagnostic journey that begins with detection of early-stage cognitive impairment with a brief cognitive test in primary care, referral to specialty care for further evaluation, and biomarker testing for cases with confirmed cognitive impairment. Wait times were projected under standard of care assumptions, i.e., no blood test available, and assuming availability of an AD blood test labeled as a “rule out” or “rule in” test in primary care. Assumptions for the effect of using the blood test on referral decisions were obtained through a structured expert consultation process. We modeled the 10-year timeframe from 2027 to 2036, for use in the US population aged 50 and older.

### Model description

The Markov model simulates the diagnostic journey of patients who are seeking evaluation for subjective memory complaints or as part of a preventive exam in primary care. It reflects the highly stylized patient journey through different evaluation stages: the standard of care evaluation is comprised of an initial evaluation by a primary care physician with a brief cognitive assessment, a comprehensive assessment by an AD specialist, and, upon confirmation of early-stage cognitive impairment, confirmatory biomarker testing with PET scanning or CSF testing. Based on input from our expert panel, we assumed that 75% of confirmatory biomarker tests would be conducted with amyloid PET scans and 25% based on CSF analysis. Irrespective of those results, all patients would proceed to a second specialist visit to devise a treatment plan. Appointments for the second specialist visit are prioritized, i.e., appointments for the first visit only become available if no patient waits for his or her second visit. The number of AD specialist appointments and PET scan slots are assumed to be capacity constrained.

The model evaluated how inserting a blood test for AD pathology into this diagnostic process would affect wait times. The test would be used in primary care after suspected early-stage cognitive impairment was detected with a brief cognitive assessment, as a triage or “rule-out” test or as a confirmatory or “rule-in” test. The model has been described in detail in earlier publications,^14,15^ and a technical description including a model schematic and model parameters are documented in Supplemental Figure 1 and Supplemental Table 1.

### Model parameters

#### Population and disease burden

Data on the US population as well as growth trends and mortality rates were obtained from US Census data. Data for incidence and prevalence of MCI by age group were obtained from prior studies by Gillis et al.^16^ and Petersen et al.,^17^ respectively, and corresponding data for mild dementia from Manly et al.^18^ and Gillis et al.^19^ The proportion of individuals with early stage cognitive impairment with AD as the underlying pathology by age came from Gustavsson et al.^20^ (Supplemental Table 2).

#### Capacity assumptions

We assumed that 22,178 dementia specialists (n = 13,569 general or behavioral neurologists, n = 1272 geriatric psychiatrists, n = 5918 geriatricians and n = 1419 general psychiatrists with a focus on dementia care were practicing in the US in 2022 based on AMA Masterfile data and each could provide on average 2860 consultations per year,^21^ of which 5% would be devoted to evaluation of patients for eligibility of amyloid-targeting treatment with an annual growth of capacity of 0.66% and 0.14%. The exact derivation is described in Supplemental Tables 3 and 4 and accompanying text.

OECD health statistics data from 2015 to 2020, the most recent year available, were used to derive current and projected future numbers of PET scans conducted in the US. In 2020, the number reached 2,220,300 scans with an annual growth rate of 4.4% (Supplemental Table 5). As device utilization is low with 1,166 scans per device in 2020 relative to an expected rate of 3,150,^22^ we assumed that 50% excess capacity could be devoted to amyloid PET scans. Detailed calculations are shown in Supplemental Figure 2 and Supplemental Table 6.

#### Diffusion model

Everett Rogers’ seminal work “Diffusion of Innovations”^23^ postulated that uptake of innovations follows an S-shaped trajectory with initial slow growth as only “Innovators” and “Early Adopters” embrace the innovation, followed by exponential growth as the “Early Majority” and then the “Late Majority” joins in and then flattening out as a residual number of “Laggards” adopt. We applied his theoretical framework to the case of diffusion of the amyloid-targeting treatments using the spread of minimally invasive coronary artery bypass surgery as an analog and leveraged published data of the proportion of adopters of electronic health records^24^ in each of the five categories to estimate the annual number of dementia specialists, who would conduct diagnostic evaluations to assess eligibility for amyloid-targeting treatments. The Supplemental Material contains a detailed description of the analysis.

#### Blood test

The published performance characteristics of the PrecivityAD2 blood test, which measures the p-tau217/np-tau217 ratio and the amyloid-β 42/40 ratio to detect amyloid PET positivity, of a sensitivity of 88% and a specificity of 89% were used for this study.^13^

#### Expert panel consultation process to derive clinical utility estimates

We conducted a structured expert consultation process with a modified Delphi approach^25^ to generate estimates for how AD blood test results would inform clinical decisions as data for real-world practice remain sparse.^26^ The expert group included five members, one geriatric psychiatrist, two neurologists, and two geriatricians from a variety of geographic areas and institutional settings. The experts were briefed on the study's approach and were then asked to provide estimates individually, assuming that all tests had regulatory approval and coverage, and that clinicians had full autonomy to make decisions based on test results.

The answers were compiled and analyzed, and results reported back to the group together with feedback and clarifications, if requested. Subsequently, the experts were given an opportunity to change their estimates, if they considered it appropriate. The median of the final ratings was considered the consensus estimate.

**Figure 1.**
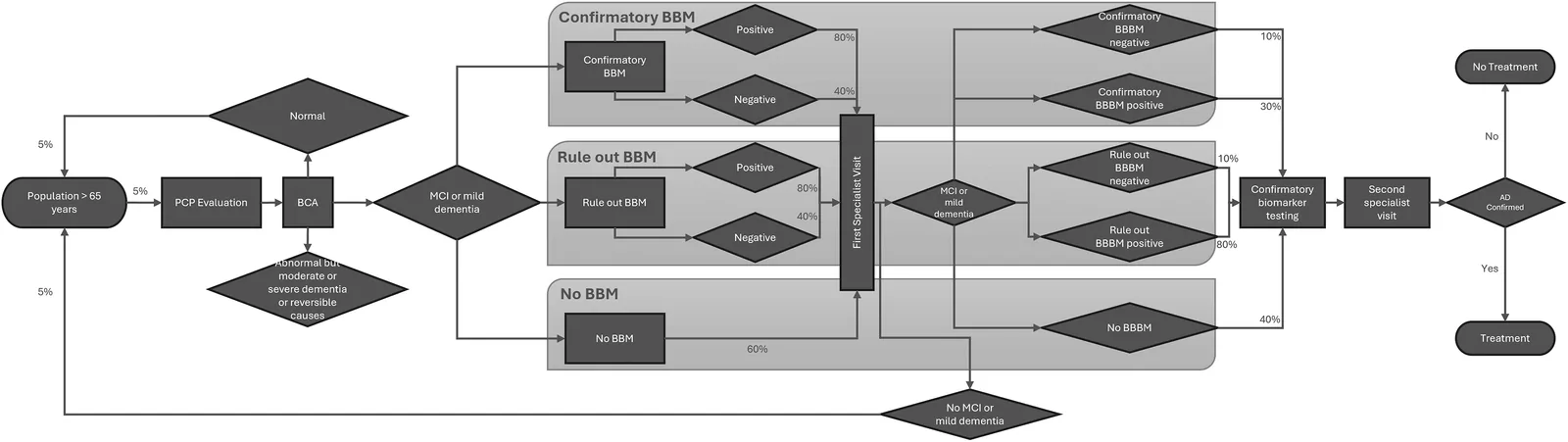
Expert assumptions for diagnostic pathway towards determination of early-stage Alzheimer's disease.

Figure 1 displays those final estimates for the three scenarios and Supplemental Table 7 the individual ratings. Of the population aged 50 and older, 5%, who never had a cognitive assessment and 5%, who previously tested cognitively normal, would see their primary care clinician for such a test each year. Under standard of care, i.e., no blood test available, primary care clinicians would refer 60% of patients with an abnormal brief cognitive test suggesting MCI or mild dementia to an AD specialist. Specialists would then conduct CSF or PET testing in 40% of patients with confirmed early-stage cognitive impairment. If a blood test were available in primary care, 80% of those with a positive test and 40% with a negative result would be referred, if their brief cognitive test also suggested MCI or mild dementia. If the blood test were labeled as triage test, specialists would conduct confirmatory testing in 80% and 10% of those with a positive and negative test, respectively. If the test were labeled as confirmatory, 30% and 10% of cases with confirmed early-stage cognitive impairment would undergo additional testing with PET or CSF. Put differently, specialists would consider a positive blood test result as sufficient evidence of AD pathology in 20% of cases, if the test were labeled as triage test, and 70%, if it were labeled as confirmatory test.

#### Analysis

We modeled the 10-year timeframe from 2027 to 2036 with the US population aged 50 and older as the intended use population. This population-level model added each year the number of individuals, who have aged into the included range, and those who previously tested negative during the evaluation process, and removed those, who have progressed to moderate or severe dementia or died.

## Results

### Capacity projections

Table 1 shows the projected number of appointments that would be available for amyloid PET scans from 2027 to 2036. The number would grow from around 1.5 million in 2027 to around 2.2 million in 2036.

**Table 1.** Projected number of available amyloid PET scan appointments.

Year	Number of amyloid PET scans
2027	1,496,882
2028	1,562,180
2029	1,630,327
2030	1,701,447
2031	1,775,670
2032	1,853,130
2033	1,933,969
2034	2,018,335
2035	2,106,381
2036	2,198,268

**Figure 2.**
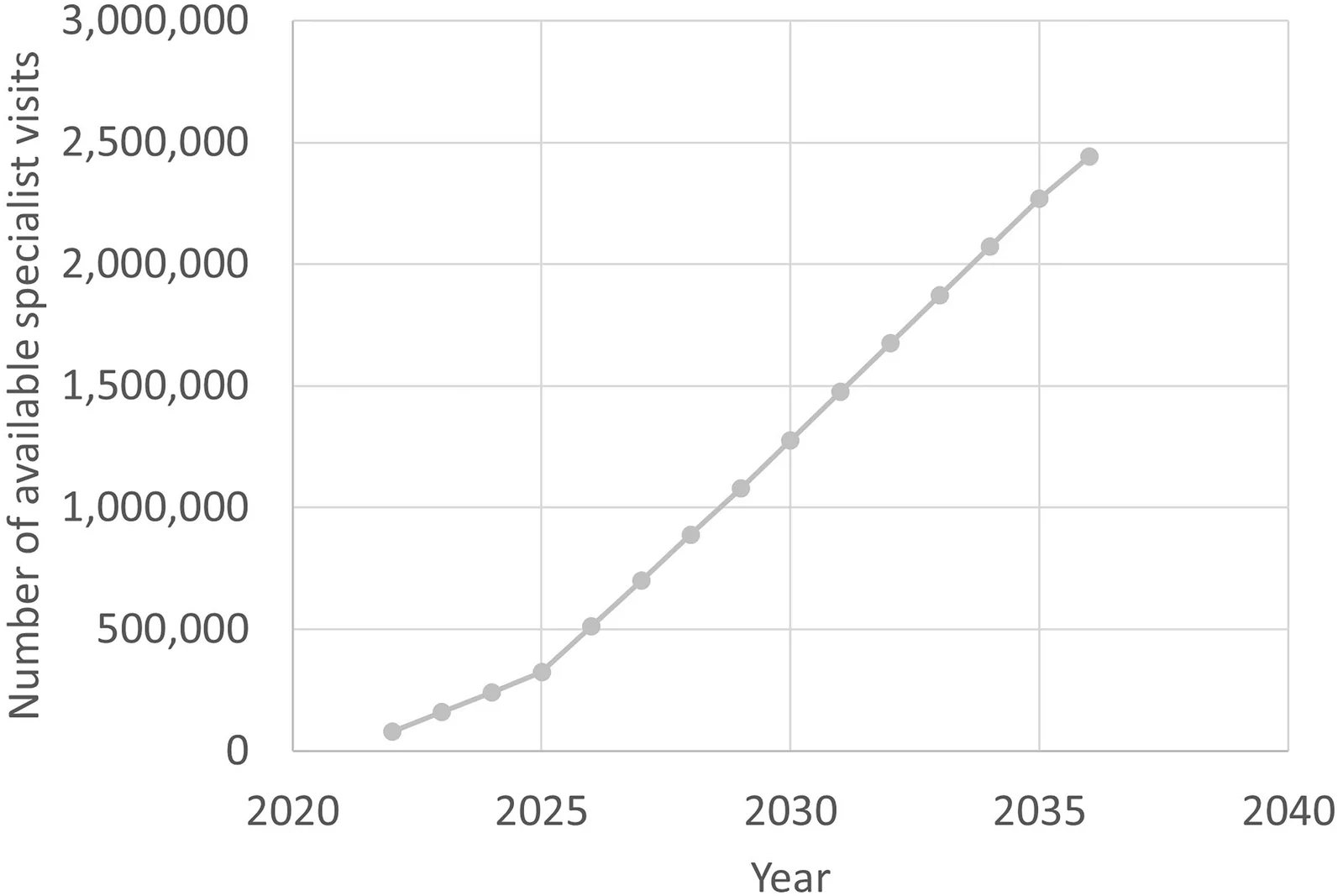
Projected availability of dementia specialist consultations.

The projected number of AD specialist appointments is illustrated in Figure 2 and detailed in the Supplemental Material. After slow growth in the years 2022 to 2025, growth accelerates as the early adopters begin offering amyloid targeting treatments. Consequently, the number of available appointments would increase from 701,053 in 2027 to 2,443,002 in 2036.

### Wait times projections

**Figure 3.**
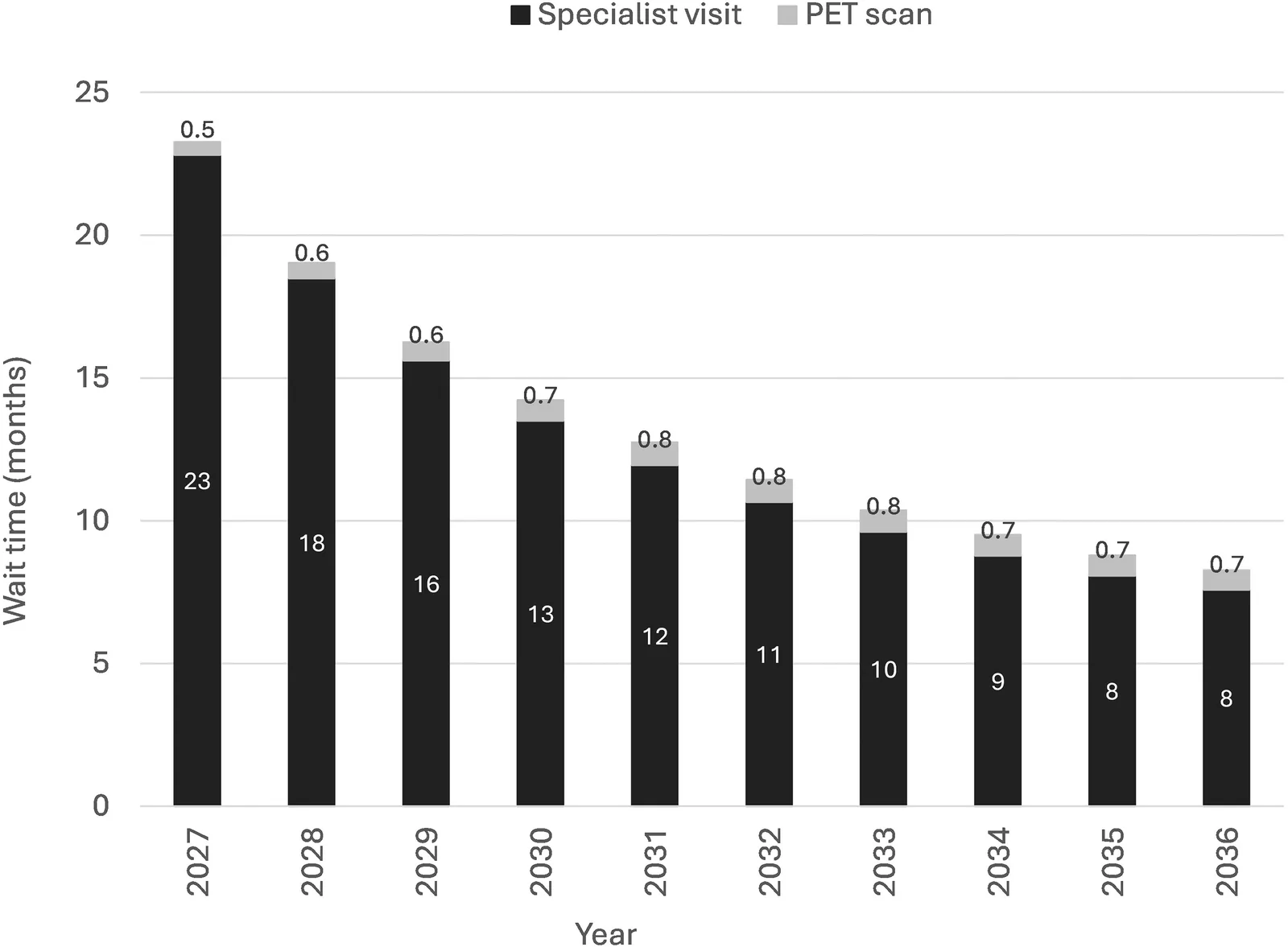
Projected wait times in the diagnostic process, referral to specialists based on results from a brief cognitive assessment only.

Figure 3 displayed estimated wait times if referral decisions to specialty were based on the results of a brief cognitive assessment. An initial gap of around 630,000 specialist appointments would translate into wait times of 23 months in 2027. With increasing specialist participation, the wait list would begin to clear in 2029 and wait times to fall to 8 months in 2031. Wait times for PET scans would initially be half a month, because patients are held up in the queue for their first specialist appointments, as evidenced by the fact that wait times are projected to increase as wait lists for specialist appointments clear.

**Figure 4.**
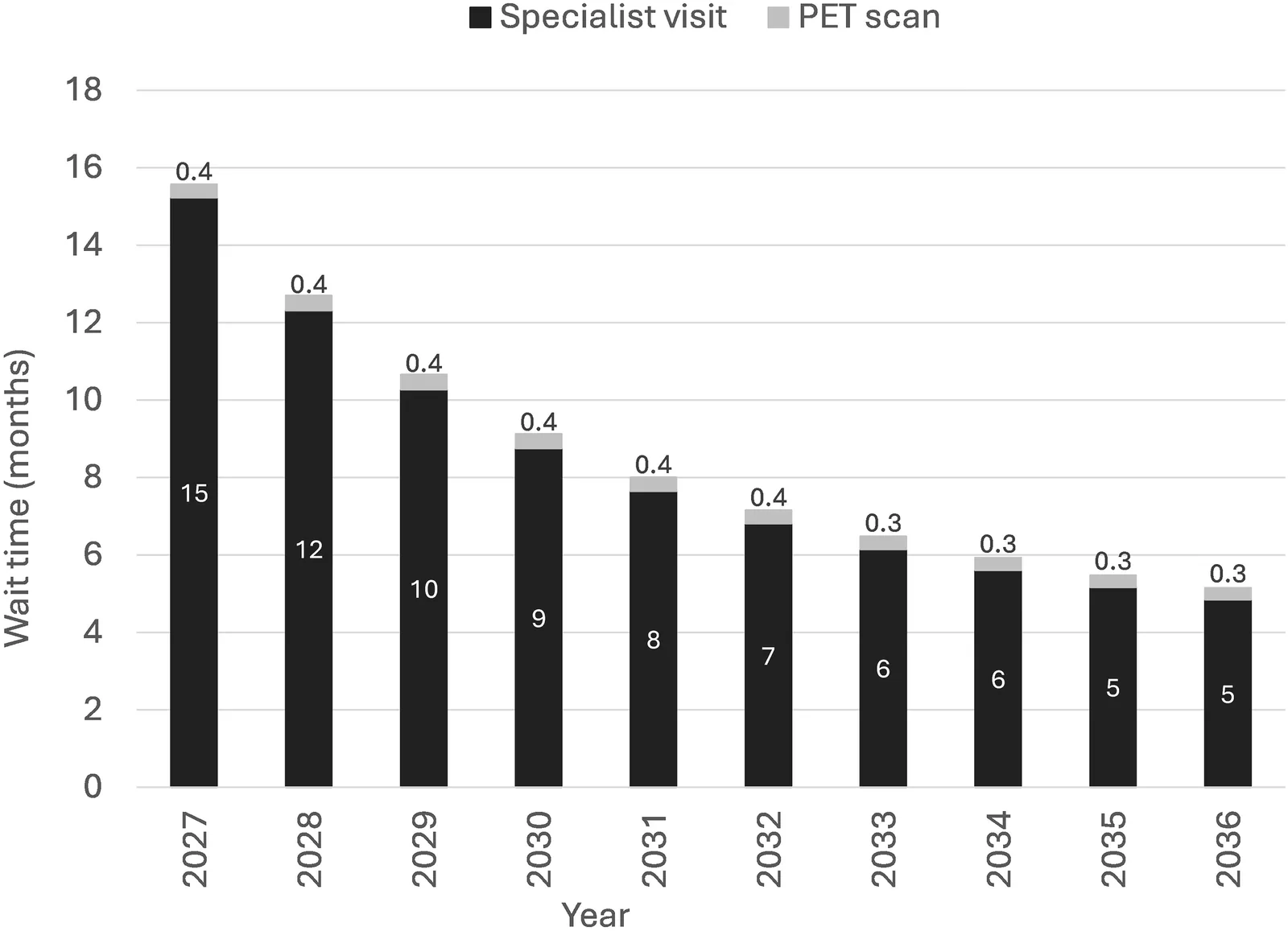
Projected wait times in the diagnostic process, referral to specialists based on results from a brief cognitive assessment and a “rule-out” blood test.

**Figure 5.**
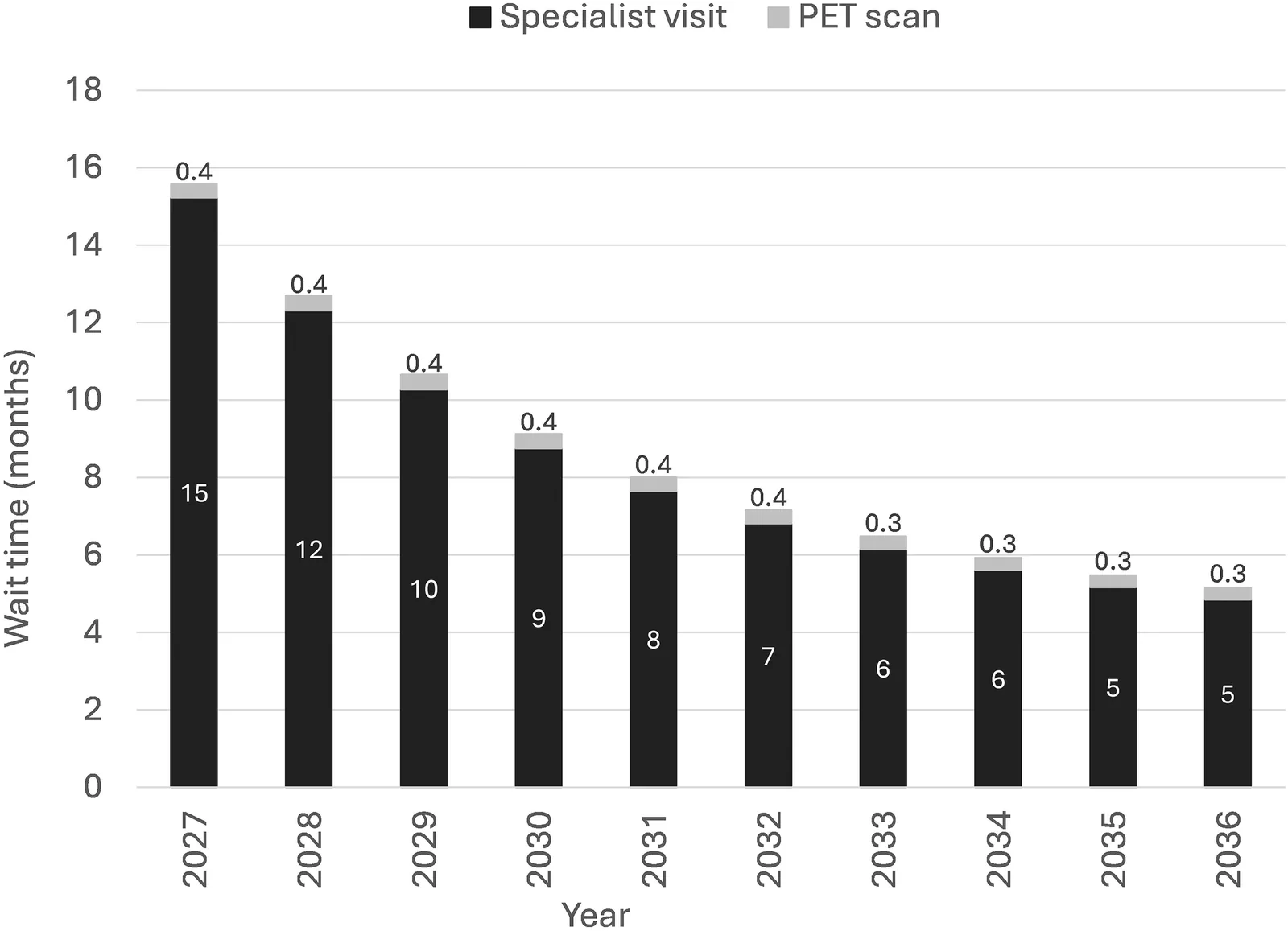
Projected wait times in the diagnostic process, referral to specialists based on results from a brief cognitive assessment and a confirmatory blood test.

The effect of using a blood test in individuals with a cognitive assessment suggesting early-stage impairment is illustrated in Figures 4 and 5. Wait times for specialist appointments are identical for blood biomarkers labeled as triage (Figure 4) and confirmatory test (Figure 5) with an estimated 15 months in 2027 falling to 5 months in 2036. As fewer patients would undergo biomarker testing with PET or CSF in the case of a confirmatory blood biomarker test, there would be virtually no wait times for amyloid PET scans, whereas wait times would be 1–2 weeks in the case of a triage blood test.

## Discussion

In this modeling study, we projected the effect of inserting a blood test for AD pathology into the initial evaluation for possible treatment with an amyloid-targeting treatment in primary care and found that wait times for AD specialist appointments would be reduced by 35%, from 23 to 15 months, compared with evaluating patients with a brief cognitive assessment only. The results are consistent with an earlier publication that the availability of blood test results increases the diagnostic accuracy^27^ of both primary and specialty care physicians, which should translate into fewer referrals. While wait times for PET scans were limited even without the use of a blood test, the reduction associated with the insertion of a blood test into the diagnostic process is comparable to two earlier clinical utility studies that both estimated a ∼50% lower rate of additional brain amyloid evaluations.^26,28^

Both wait times and the estimated effect of a blood test are less pronounced but are more closely aligned with current anecdotal reports^7^ than in an earlier publication,^15^ which assumed much higher cognitive testing rates in primary care and referral decisions that we solely based on diagnostic test results. In contrast, the current study uses expert assumptions for how test results would inform rather than determine clinical decisions, which results in much lower referral rates to specialist appointments and biomarker testing with PET scans and CSF analysis and thus wait times. The assumption of a low cognitive testing rate in primary care was informed by data on the consistently low use of formal cognitive tests during the Medicare Annual Wellness Visit even in higher-risk individuals.^29^ It should not go unnoticed, however, that cognitively impaired individuals who are not evaluated might experience disease progression, even though they do not appear on the wait lists. Thus, the modest and rapidly falling projected wait times should not be seen as reassuring but as a symptom of the underlying low detection rate of early-stage cognitive impairment in primary care: Studies have estimated that only 8–10% of MCI cases in the elderly Medicare population are currently diagnosed,^9,30^ and efforts to increase the detection rate are urgently needed. The success of such efforts would in turn heighten the needs for scalable and automated tests in the evaluation of cognitive impairment, not just for AD pathology, but also for cognitive assessment.^31^ Higher detection rate would in turn require efforts to avoid longer wait times. Ensuring a timely evaluation of treatment eligibility is critical, as recent data from the open-label extension study of lecanemab, one of the two treatments available in the US, suggest that the treatment can stop disease progression entirely and even improve cognition and functions when used in earliest disease states, identified as no or low tau burden.^32^ Further, additional treatments might become available in the near future that would broaden the range of eligible patients.^33^

### Limitations

We need to acknowledge the limitations of this study. First and foremost, modeling studies do not represent direct evidence, and their results need to be validated with real-world data, especially if they use such a highly stylized patient journey. The same goes for the assumed effects of blood test availability on physician decisions that we based on expert input and the assumption that patients would follow the recommended diagnostic pathway. While the experts showed convergence for decisions assuming availability of blood test results, there was greater variability in estimates for decisions in the absence of a blood test, which suggest differences in practice style. We assumed that the blood test would be used in primary care, which is currently not guideline-recommended.^34^ Other treatments may emerge during the projection period^33^ which could alter wait times and the estimated effect of the blood test. Accordingly, the projection should be understood as illustrating the scale of the challenge and the relative impact of blood biomarkers, rather than as a precise quantitative estimate. Lastly, the estimates represent US averages and may not generalize to subnational areas and other countries.

### Conclusions

Even with the currently low detection rates of early-stage cognitive impairment, wait times in the diagnostic process for eligibility for amyloid-targeting treatments in AD can be almost two years under current standard of care. As the use of blood tests for the AD pathology is projected to reduce those wait times substantially, regulatory approval and coverage of those tests as well as guideline recommendations for their use in primary care are urgently needed, especially if detection rates of early-stage cognitive impairment are increasing.

## Supplemental Material

sj-docx-1-alz-10.1177_13872877261467345 - Supplemental material for Projected effect of a high performing blood test on wait times for determination of Alzheimer's disease pathology in the United States Supplemental material, sj-docx-1-alz-10.1177_13872877261467345 for Projected effect of a high performing blood test on wait times for determination of Alzheimer's disease pathology in the United States by Soeren Mattke, Jiahe Chen, Mark Hanson, Kim G. Johnson, Cara Leahy, David A. Merrill, Victoria Shada and Jorge G. Ruiz in Journal of Alzheimer's Disease
