# Supplementary material for: Projected effect of a high performing blood test on wait times for determination of Alzheimer's disease pathology in the United States
Source: J Alzheimers Dis. 2026 Jul 16;113(1):177–85. doi: 10.1177/13872877261467345 (PMC13499904; doi:10.1177/13872877261467345)
Supplement: Supplementary file 1 [file sj-docx-1-alz-10.1177_13872877261467345.docx]

# **Supplemental Material**

**Projected effect of a high performing blood test on wait times for determination of Alzheimer’s disease pathology in the United States**

## **Supplemental Table 1.** Model input parameters

|  | **Value** | **Source** |  |
| --- | --- | --- | --- |
| Hazard ratio for excess mortality | | | |
| MCI | 1.43 | ^1^ |  |
| Dementia | 3.26 | ^2,3^ |  |
| Annual transition probability | | | |
| Cognitively normal to MCI | 0.030 | ^4^ |  |
| MCI to mild dementia | 0.065 | ^5^ |  |
| Initial and confirmatory tests | | | |
| MMSE – Sensitivity | 0.82 | ^6^ |  |
| MMSE – Specificity | 0.73 |  |  |
| Blood biomarker test – Sensitivity | 0.88 | ^7^ |  |
| Blood biomarker test – Specificity | 0.89 |  |  |
| Confirmatory cognitive testing – Sensitivity | 0.95 | Assumption |  |
| Confirmatory cognitive testing – Specificity | 0.95 | Assumption |  |
| Confirmatory testing with CSF – Sensitivity | 0.91 | ^8^ |  |
| Confirmatory testing with CSF – Specificity | 0.89 |  |  |
| Confirmatory testing with PET – Sensitivity | 0.92 | ^9^ |  |
| Confirmatory testing with PET – Specificity | 0.95 |  |  |
| Proportion of patients receiving amyloid PET scan | 75% | Expert input |  |

## **Supplemental Figure 1.** Schematic description of the model

## **Supplemental Table 2.** Disease prevalence estimates

|  | Male |  |  |  |  |  |  |  |  |
| --- | --- | --- | --- | --- | --- | --- | --- | --- | --- |
|  | **50-54** | **55-59** | **60-64** | **65-69** | **70-74** | **75-79** | **80-84** | **>= 85** | **Source** |
| MCI | 3.21% | 4.43% | 6.70% | 8.40% | 10.10% | 14.80% | 25.20% | 30.53% | ^10^ |
| mild DE | 0.04% | 0.15% | 0.39% | 1.11% | 1.48% | 5.20% | 10.40% | 10.95% | ^11,12^ |
| mod/sev DE | 0.40% | 0.67% | 1.09% | 1.89% | 2.52% | 3.80% | 7.60% | 17.05% | ^11^ |
|  |  |  |  |  |  |  |  |  |  |
| MCI due to AD | 0.91% | 1.44% | 2.70% | 3.80% | 5.20% | 8.50% | 15.90% | 25.80% | ^13^ |
| mild DE due to AD | 0.01% | 0.05% | 0.16% | 0.50% | 0.76% | 2.99% | 6.56% | 9.25% | ^13^ |
|  |  |  |  |  |  |  |  |  |  |
|  |  |  |  |  |  |  |  |  |  |
|  | Female |  |  |  |  |  |  |  |  |
|  | 50-54 | 55-59 | 60-64 | 65-69 | 70-74 | 75-79 | 80-84 | >= 85 |  |
| MCI | 3.21% | 4.43% | 6.70% | 8.40% | 10.10% | 14.80% | 25.20% | 30.53% | ^10^ |
| mild DE | 0.04% | 0.15% | 0.39% | 1.11% | 1.48% | 5.20% | 10.40% | 10.95% | ^11,12^ |
| mod/sev DE | 0.40% | 0.67% | 1.09% | 1.89% | 2.52% | 3.80% | 7.60% | 17.05% | ^11^ |
|  |  |  |  |  |  |  |  |  |  |
| MCI due to AD | 0.91% | 1.44% | 2.70% | 3.80% | 5.20% | 8.50% | 15.90% | 25.80% | ^13^ |
| mild DE due to AD | 0.01% | 0.05% | 0.16% | 0.50% | 0.76% | 2.99% | 6.56% | 9.25% | ^13^ |

MCI: mild cognitive impairment; DE: dementia; mod: moderate; sev: severe; AD: Alzheimer’s disease. Numbers in red are based on exponential extrapolation

## *Specialist capacity*

Based on the 2022 AMA Masterfile, we identified all physicians specializing in general or behavioral neurology (n=13,569), geriatric psychiatry (n=1,272) and geriatrics (n=5,918) as dementia specialists. In addition, we considered general psychiatrists as dementia specialists if they had at least 10 encounters or 10% of their overall encounters with Medicare patients for a primary diagnosis of dementia or mild cognitive impairment in all three years from 2019 to 2021 (n=1419) for a total of 22,178 dementia specialists.

According to a neurology workforce projection, neurologists have on average 42.3 weekly working hours and provide on average 2,860 consultations per year.^14^ The estimate for consultations is higher than that for Germany^15^ with 2,294 consultations in specialist practices and 1,644 in hospitals, England^16^ with 1,776 consultations and Sweden^17^ with 1,130 consultations. Based on a prior study by Liu et al., we assumed that on average 5% of these consultations would be available for the evaluation of AD cases and that net capacity would grow by 0.67% annually.^18^

## *Diffusion model*

Everett Rogers' seminal work “Diffusion of Innovations”^19^ postulated that uptake of innovations follows an S-shaped trajectory with initial slow growth as only Innovators and Early Adopters embrace the innovation, followed by exponential growth as the Early and then Late Majority joins in and then flattening out as a residual number of Laggards adopt. We used the mid-point estimates for the proportion of each adoption group by Poon et al., who analyzed uptake of electronic health records, for our estimates,^20^ and derived an estimate for the duration of each adoption phase in healthcare from the case of minimally invasive coronary artery bypass surgery (MIDCAP). MIDCAP originated in Stanford in 1994^21^ and spread to some large academic hospitals in the late 1990s.^22^ In the early years of the new millennium, accelerated adoption took off^23^ and the 2004 American College of Cardiology/American Heart Association guideline^24^ made it an officially sanctioned mainstream procedure that became routine practice in the 2010s.^21^

MIDCAP is a suitable analog for the introduction of the amyloid-targeting treatments, as it represented a paradigm shift over open-heart surgery with substantial requirements for surgeon training, capital investment, evidence generation and patient education. We chose 2022, the first calendar year after the approval of aducanumab, as our starting year for the diffusion of amyloid-targeting treatments and projected the spread of adoption following a study on diffusion of electronic health records by Poon et al.^20^ (Supplemental Table 3) We applied the proportion of adopters in each diffusion phase and the duration of each phase to the number of dementia specialists, who would conduct diagnostic evaluations of potential eligibility for amyloid-targeting treatments, as they represent the most critical bottleneck in the patient journey.

## **Supplemental Table 3.** Diffusion projection for amyloid-targeting treatments

| Diffusion phase | Proportion of adopters | Midpoint of proportion of adopters | Starting year MIDCAP | Starting year ATT |
| --- | --- | --- | --- | --- |
| Innovators | 0–5% | 2.5% | 1994 | 2022 |
| Early Adopters | 5–15% | 10% | 1997 | 2025 |
| Early Majority | 15–50% | 33% | 2001 | 2029 |
| Late Majority | 50–85% | 68% | 2007 | 2035 |
| Widespread Adoption | 85–100% | 93% | 2012 | 2040 |

MIDCAP denotes minimally invasive coronary artery bypass surgery, ATT denotes amyloid-targeting treatments. Data for proportion of adopters are from Poon et al. and for the starting years based on authors’ analysis

Applying these diffusion estimates to the estimated numbers of dementia specialists yields the annual number of visits that are available for diagnosis of AD patients (Supplemental Table 4).

## **Supplemental Table 4.** Projected number of available dementia specialist visits

| Year | Number of dementia specialists | Adopters among dementia specialists | Total number of consultations available from adopters | Total number of consultations available for evaluation of AD treatment eligibility |
| --- | --- | --- | --- | --- |
| 2022 | 22,178 | 554 | 1,585,727 | 79,286 |
| 2023 | 22,325 | 1,124 | 3,213,581 | 160,679 |
| 2024 | 22,473 | 1,693 | 4,841,435 | 242,072 |
| 2025 | 22,620 | 2,262 | 6,469,289 | 323,464 |
| 2026 | 22,767 | 3,582 | 10,245,176 | 512,259 |
| 2027 | 22,914 | 4,902 | 14,021,063 | 701,053 |
| 2028 | 23,062 | 6,223 | 17,796,950 | 889,847 |
| 2029 | 23,209 | 7,543 | 21,572,837 | 1,078,642 |
| 2030 | 23,356 | 8,932 | 25,545,510 | 1,277,276 |
| 2031 | 23,389 | 10,321 | 29,518,184 | 1,475,909 |
| 2032 | 23,423 | 11,710 | 33,490,857 | 1,674,543 |
| 2033 | 23,456 | 13,099 | 37,463,530 | 1,873,177 |
| 2034 | 23,489 | 14,488 | 41,436,204 | 2,071,810 |
| 2035 | 23,522 | 15,877 | 45,408,877 | 2,270,444 |
| 2036 | 23,555 | 17,084 | 48,860,036 | 2,443,002 |

## *Capacity for amyloid PET scans*

Table A.5 shows data from OECD Health Statistics ^25^ for the annual number of PET scans in all settings from 2015 to 2020, which is most recent year available.

## **Supplemental Table 5.** Annual number of PET scans performed in the U.S.

| Year | Number of PET scans |
| --- | --- |
| 2015 | 1,718,500 |
| 2016 | 1,873,900 |
| 2017 | 1,945,000 |
| 2018 | 2,086,000 |
| 2019 | 2,200,800 |
| 2020 | 2,220,300 |

As Supplemental Figure 2 illustrates, the reported numbers suggest a linear growth trend with a Compound Annual Growth Rate of 4.4%.

## **Supplemental Figure 2.** Annual growth of number of PET scans performed in the U.S.

Number of Scans

|  | Number |
| --- | --- |
| 2015 | 1718500 |
| 2016 | 1873900 |
| 2017 | 1945000 |
| 2018 | 2086000 |
| 2019 | 2200800 |
| 2020 | 2220300 |

Also, according to OECD data^25^ (Supplemental Table 2), the number of scans per device is almost flat during this period at a comparatively low utilization rate. Utilization rates for other G7 countries are considerably higher with a range of 2,000 in Sweden ^17^ to 3,280 for the NHS England.^16^ Similarly Wittenberg et al.^26^ estimated that a PET scanner can accommodate around 3,125 scans per year. Thus, installed devices in the U.S. are not run at full capacity, and we assume that the installed base would permit conducting 50% (40-60%) additional scans per year which would be devoted to amyloid PET with an annual 4.4% growth of capacity.

## **Supplemental Table 6.** Average number of PET scans per device

| Year | Scans per device |
| --- | --- |
| 2015 | 1,045 |
| 2018 | 1,216 |
| 2019 | 1,230 |
| 2020 | 1,166 |

## **Supplemental Table 7.** Individual expert rating results and analysis of distribution

| **Question** | | **R1** | **R2** | **R3** | **R4** | **R5** |  | **Median** | **Mean absolute difference from median** |
| --- | --- | --- | --- | --- | --- | --- | --- | --- | --- |
| **Screening age** | |  |  |  |  |  |  |  |  |
| At which age would screening for cognitive impairment in primary care start, assuming it became a covered benefit? | | 70 | 65 | 65 | 65 | 55 |  | 65 | 14 |
| Would there be an upper age limit for screening? | | No | Yes | No | No | No |  | No |  |
| If you stated that there would be an upper age limit, please enter it here: | | N/A | 85 | N/A | N/A | N/A |  | N/A |  |
| **Primary care assessment** | |  |  |  |  |  |  |  |  |
| What proportion of individuals, who have **never been tested for cognitive impairmen**t, would seek a brief cognitive assessment (e.g., MMSE or MoCA) in primary care in a given year? | | 15% | 20% | 30% | 18% | 20% |  | 20% | 7% |
| What proportion of individuals, who have **previously tested as cognitively normal**, would seek a brief repeat assessment if primary care each year? | | 7% | 10% | 30% | 30% | 10% |  | 10% | 11% |
| If a patient were negative on a brief cognitive test, how many years, on average, would pass until they get retested, assuming they are not developing new subjective memory concerns? | | 2 | 3 | 3 | 3 | 2 |  | 3 | 0.80 |
| No Blood Test Available | What proportion of individuals, whose brief cognitive assessment suggests MCI or mild dementia, would be **referred to a specialist** for further evaluation, assuming that **no blood test for the AD patholog**y is available? | 70% | 30% | 50% | 60% | 60% |  | 60% | 10% |
| **Blood Test available:** Assume a blood test for the AD pathology with a sensitivity of 88% and a specificity of 89% | What proportion of individuals, whose brief cognitive assessment suggests MCI or mild dementia, and have a **blood test suggestive of the AD pathology** would be referred to a specialist for further evaluation? | 80% | 60% | 60% | 80% | 90% |  | 80% | 10% |
|  | What proportion of individuals, whose brief cognitive assessment suggests MCI or mild dementia, and have a **blood test NOT suggestive of the AD pathology** would be referred to a specialist for further evaluation? | 70% | 10% | 30% | 40% | 50% |  | 40% | 16% |
| **Specialist assessment** | |  |  |  |  |  |  |  |  |
| No Blood Test Available | Consider the individuals, in whom the specialist has confirmed the presence of MCI or mild dementia: What proportion of those who did not have a blood test, would be sent to confirmatory biomarker testing with CSF or PET? | 40% | 75% | 10% | 70% | 30% |  | 40% | 21% |
| **Rule-out Blood Test** with a sensitivity of 88% and a specificity of 89% | Proportion of individuals, in whom the AD specialist performs a comprehensive assessment, including neurocognitive testing to confirm early-stage cognitive impairment, in the first visit and are referred to **biomarker testing with PET or CSF** for the AD pathology, assuming a **positive blood test** that is labeled as rule out test | 80% | 80% | 75% | 90% | 90% |  | 80% | 5% |
|  | Proportion of individuals, in whom the AD specialist performs a comprehensive assessment, including neurocognitive testing to confirm early-stage cognitive impairment, in the first visit and are **referred to biomarker testing with PET or CSF** for the AD pathology, assuming a **negative blood test** that is labeled as rule out test | 15% | 10% | 2% | 10% | 2% |  | 10% | 4% |
| **Rule-in Blood Test:** Assume a blood test for the AD pathology with a sensitivity of 88% and a specificity of 89% that is labeled and reimbursed as confirmatory test | What proportion of patients with specialist-confirmed early-stage cognitive impairment, who also had a **positive blood test**, would be diagnosed with early-stage AD without further confirmatory biomarker testing? | 26% | 30% | 10% | 30% | 40% |  | 30% | 7% |
|  | What proportion of patients with specialist-confirmed early-stage cognitive impairment, who also had a **negative blood test**, would undergo confirmatory biomarker testing? | 15% | 10% | 2% | 10% | 10% |  | 10% | 3% |
| Consider the individuals, in whom the specialist has confirmed the presence of MCI or mild dementia: If confirmatory biomarker testing were conducted, **what proportion would be performed with CSF analysis**? | | 20% | 25% | 5% | 50% | 25% |  | 25% | 10% |

## **References**

1.	Vassilaki M, Cha RH, Aakre JA, et al. Mortality in mild cognitive impairment varies by subtype, sex, and lifestyle factors: the Mayo Clinic Study of Aging. *J Alzheimers Dis* 2015; 45: 1237-1245.

2.	Neumann PJ, Araki SS, Arcelus A, et al. Measuring Alzheimer's disease progression with transition probabilities: estimates from CERAD. *Neurology* 2001; 57: 957-964.

3.	Murphy SL, Xu J, Kochanek KD, et al. Mortality in the United States, 2017. *NCHS Data Brief* 2018; (328): 1-8.

4.	Yesavage JA, O’Hara R, Kraemer H, et al. Modeling the prevalence and incidence of Alzheimer’s disease and mild cognitive impairment. *J Psychiatr Res* 2002; 36: 281-286.

5.	Mitchell AJ and Shiri-Feshki M. Rate of progression of mild cognitive impairment to dementia - meta-analysis of 41 robust inception cohort studies. *Acta Psychiatr Scand* 2009; 119: 252-265.

6.	Roalf DR, Moberg PJ, Xie SX, et al. Comparative accuracies of two common screening instruments for classification of Alzheimer's disease, mild cognitive impairment, and healthy aging. *Alzheimers Dement* 2013; 9: 529-537.

7.	Meyer MR, Kirmess KM, Eastwood S, et al. Clinical validation of the PrecivityAD2 blood test: A mass spectrometry‐based test with algorithm combining %p‐tau217 and Aβ42/40 ratio to identify presence of brain amyloid. *Alzheimers Dement* 2024; 20: 3179-3192.

8.	Hansson O, Seibyl J, Stomrud E, et al. CSF biomarkers of Alzheimer's disease concord with amyloid-beta PET and predict clinical progression: A study of fully automated immunoassays in BioFINDER and ADNI cohorts. *Alzheimers Dement* 2018; 14: 1470-1481.

9.	Clark CM, Pontecorvo MJ, Beach TG, et al. Cerebral PET with florbetapir compared with neuropathology at autopsy for detection of neuritic amyloid-beta plaques: a prospective cohort study. *Lancet Neurol* 2012; 11: 669-678.

10.	Petersen RC, Lopez O, Armstrong MJ, et al. Practice guideline update summary: Mild cognitive impairment. *Neurology* 2018; 90: 126-135.

11.	Manly JJ, Jones RN, Langa KM, et al. Estimating the prevalence of dementia and mild cognitive impairment in the US: The 2016 Health and Retirement Study Harmonized Cognitive Assessment Protocol Project. *JAMA Neurol* 2022; 79: 1242-1249.

12.	Gillis C, Montenigro P, Nejati M, et al. Estimating prevalence of early Alzheimer's disease in the United States, accounting for racial and ethnic diversity. *Alzheimers Dement* 2023; 19: 1841-1848.

13.	Gustavsson A, Norton N, Fast T, et al. Global estimates on the number of persons across the Alzheimer's disease continuum. *Alzheimers Dement* 2023; 19: 658-670.

14.	Dall TM, Storm MV, Chakrabarti R, et al. Supply and demand analysis of the current and future US neurology workforce. *Neurology* 2013; 81: 470-478.

15.	Mattke S, Tang Y, Hanson M, et al. Current capacity for diagnosing Alzheimer's disease in Germany and implications for wait times. *J Alzheimers Dis* 2024; 101: 1249-1259.

16.	Mattke S, Shi Z, Hanson M, et al. Estimated investment need to increase england’s capacity to diagnose eligibility for an Alzheimer’s treatment to G7 average capacity levels. *J Prev Alzheimers Dis* 2024; 11: 1022-1029.

17.	Mattke S, Gustavsson A, Jacobs L, et al. Estimates of current capacity for diagnosing Alzheimer's disease in Sweden and the need to expand specialist numbers. *J Prev Alzheimers Dis* 2024; 11: 155-161.

18.	Liu JL, Hlavka JP, Hillestad R, et al. *Assessing the Preparedness of the U.S. Health Care System Infrastructure for an Alzheimer's Treatment*. RAND Corporation, 2017.

19.	Rogers EM. Diffusion of innovations. New product adoption and diffusion. *J Consum Res* 1976; 2: 290-301.

20.	Poon EG, Jha AK, Christino M, et al. Assessing the level of healthcare information technology adoption in the United States: a snapshot. *BMC Med Inform Decis Mak* 2006; 6: 1.

21.	Chitwood Jr WR. Historical evolution of robot-assisted cardiac surgery: a 25-year journey. *Ann Cardiothorac Surg* 2022; 11: 564-582.

22.	Ribakove GH, Miller JS, Anderson RV, et al. Minimally invasive port-access coronary artery bypass grafting with early angiographic follow-up: Initial clinical experience. *J Thorac Cardiovasc Surg* 1998; 115: 1101-1110.

23.	Bonatti J. Historical landmarks in the development of robotic coronary bypass grafting. *Ann Cardiothorac Surg* 2024; 13: 332-338.

24.	Eagle KA, Guyton RA, Davidoff R, et al. ACC/AHA 2004 guideline update for coronary artery bypass graft surgery: summary article: a report of the American College of Cardiology/American Heart Association Task Force on Practice Guidelines (Committee to Update the 1999 Guidelines for Coronary Artery Bypass Graft Surgery). *Circulation* 2004; 110: 1168-1176. Erratum in: *Circulation* 2005; 111: 2014.

25.	OECD. Data from: OECD Health Statistics 2022. 2023.

26.	Wittenberg R, Knapp M, Karagiannidou M, et al. Economic impacts of introducing diagnostics for mild cognitive impairment Alzheimer's disease patients. *Alzheimers Dement (N Y)* 2019; 5: 382-387.
